# Identification of and Surveillance for the SARS-CoV-2 Variants B.1.427 and B.1.429 — Colorado, January–March 2021

**DOI:** 10.15585/mmwr.mm7019e2

**Published:** 2021-05-14

**Authors:** Lindsey Martin Webb, Shannon Matzinger, Christopher Grano, Breanna Kawasaki, Ginger Stringer, Laura Bankers, Rachel Herlihy

**Affiliations:** 1 Colorado Department of Public Health and Environment.

The B.1.427 and B.1.429 variants of SARS-CoV-2, the virus that causes COVID-19, were first described in Southern California on January 20, 2021 (*1*); on March 16 they were designated variants of concern* (*2*). Data on these variants are limited, but initial reports suggest that, compared with other lineages, they might be more infectious (*1*,*2*), cause more severe illness (*2*), and be less susceptible to neutralizing monoclonal antibody products such as bamlanivimab, an investigational treatment for mild-to-moderate COVID-19 (*1*–*3*). On January 24, the Colorado Department of Public Health and Environment (CDPHE) identified the first Colorado case of COVID-19 attributed to these variants. B.1.427 and B.1.429 were considered a single variant described as CAL.20C or B.1.427/B.1.429 in the 20C clade (*1*,*3*); in this report “B.1.427/B.1.429” refers to B.1.427 or B.1.429 lineage, including those reported as B.1.427/B.1.429 without further differentiation.

In Colorado, most routine SARS-CoV-2 whole genome sequencing (WGS) is performed by the CDPHE laboratory, generally on a convenience sample of available specimens. Whereas reverse transcription–polymerase chain reaction (RT-PCR) S-gene target failure, which suggests the presence of the SARS-CoV-2 B.1.1.7 variant, is used to prioritize specimens for sequencing (*4*), no such indicator exists for B.1.427/B.1.429 and other variants of concern. To improve convenience sampling to identify and track emerging variants, CDPHE established a 30-site statewide sentinel surveillance system. Sites submit a random sample of up to 30 SARS-CoV-2 RT-PCR–positive specimens from inpatients and outpatients to CDPHE for sequencing each week. COVID-19 B.1.427/B.1.429 variant cases were identified through tiled amplicon WGS.^†^^,^^§^ Assembly of sequencing data into whole genomes was performed using CDPHE’s publicly available Illumina and Nanopore data workflows.^¶^ Phylogenetic Assignment of Named Global Outbreak Lineages (Pangolin)** (*5*) and Nextstrain’s Nextclade tools (*6*) were used to assign lineage designations to each assembled genome. CDPHE conducted enhanced case investigation and contact tracing, including reinterview of previously interviewed persons, upstream contact tracing, increased testing of asymptomatic contacts, resource coordination to assist persons with successful isolation or quarantine, and involvement of CDPHE’s Cultural Navigation program, which ensures culturally informed communication with immigrants, refugees, and other groups that are disproportionally affected by COVID-19.^††^

By March 31, CDPHE reported 327 COVID-19 B.1.427/B.1.429 cases with specimen collection dates during January 4–March 20, including 90 (28%) B.1.427, 218 (67%) B.1.429, and 19 (6%) not differentiated by the reporting commercial laboratory. B.1.427/B.1.429 case sequences were identified a median of 14.5 days after specimen collection (range = 7–38 days). Median patient age was 39 years (range = <1–95 years); 186 (57%) patients were male. Cases were identified in 31 (48%) of Colorado’s 64 counties. Enhanced interviewing of all patients with variant cases was attempted through February; 60 (83%) such interviews were completed. Among these, nine (15%) persons reported travel outside Colorado (three to California, two to Nevada, and one each to Georgia, Minnesota, Utah, and the District of Columbia); none reported international travel. Through March, among 211 patients with symptom information available, 193 (91%) were symptomatic. Forty-six (14%) hospitalizations and eight (2%) deaths were reported; not all ill persons had recovered at time of data analysis. Based on available data, seven (2%) vaccine breakthrough cases^§§^ were identified. Although Colorado variant data were derived from a convenience sample, when compared with national estimates of 85% symptomatic illness and 5% hospitalization rates among patients with positive SARS-CoV-2 test results,^¶¶^ these data suggest that B.1.427/B.1.429 might more frequently cause discernible and severe illness than do nationally circulating lineages overall.

CDPHE tracked a steady increase in the proportion of sequenced specimens that were B.1.427/B.1.429, from 3%–4% in late January to 20%–22% in early March; during this time, national genomic surveillance data were insufficient to provide variant prevalence estimates for Colorado. Although sequencing performed for surveillance or research should not be used for individual clinical decision-making, these statewide population-level data provided general treatment decision support to clinicians for patients with positive SARS-CoV-2 test results in Colorado. Because of the increasing proportion of Colorado B.1.427/B.1.429 variant cases and their association with resistance to bamlanivimab, CDPHE issued a Health Alert Network advisory on March 22 recommending against monotherapy with bamlanivimab. On April 16, the Food and Drug Administration revoked the Emergency Use Authorization for monotherapy with this product.*** Establishing a state public health laboratory–based sequencing program and sentinel surveillance system in Colorado and merging laboratory and epidemiologic data has improved SARS-CoV-2 variant situational awareness and efforts to control the spread of variants, and also has provided data to guide Colorado clinicians and contributed timely data to inform important national clinical policy decisions. Given delays in sequencing results and increasing proportions of variant cases, all COVID-19 cases should be considered potential variant cases upon initial report.

## Footnotes

* A variant for which there is evidence of increased transmissibility, more severe disease, reduction in neutralization by vaccine- or infection-induced antibodies, reduced effectiveness of treatments or vaccines, or diagnostic detection failures.

†
https://artic.network/ncov-2019

§
https://www.protocols.io/view/sars-cov-2-sequencing-on-illumina-miseq-using-arti-bffyjjpw

¶
https://github.com/CDPHE

**
https://pangolin.cog-uk.io/

††
https://sites.google.com/state.co.us/refugeecoe/resources/cultural-navigation-2-0

§§
https://www.cdc.gov/vaccines/covid-19/health-departments/breakthrough-cases.html

¶¶
https://www.cdc.gov/coronavirus/2019-ncov/cases-updates/burden.html

***
https://www.fda.gov/news-events/press-announcements/coronavirus-covid-19-update-fda-revokes-emergency-use-authorization-monoclonal-antibody-bamlanivimab
